# Developmental expression of the receptor for advanced glycation end-products (RAGE) and its response to hyperoxia in the neonatal rat lung

**DOI:** 10.1186/1471-213X-7-15

**Published:** 2007-03-07

**Authors:** Pierre-Paul Lizotte, Lana E Hanford, Jan J Enghild, Eva Nozik-Grayck, Brenda-Louise Giles, Tim D Oury

**Affiliations:** 1 Biology of Breathing Research, Manitoba Institute of Child Health, Winnipeg, MB, Canada; 2 Department of Pathology, University of Pittsburgh, Pittsburgh, PA, USA; 3 Department of Molecular Biology, University of Aarhus, Aarhus C, Denmark; 4 Department of Pediatrics, University of Colorado, Denver, CO, USA; 5 Department of Pediatrics & Child Health and of Physiology, University of Manitoba, Winnipeg, MB, Canada

## Abstract

**Background:**

The receptor for advanced glycation end products (mRAGE) is associated with pathology in most tissues, while its soluble form (sRAGE) acts as a decoy receptor. The adult lung is unique in that it expresses high amounts of RAGE under normal conditions while other tissues express low amounts normally and up-regulate RAGE during pathologic processes. We sought to determine the regulation of the soluble and membrane isoforms of RAGE in the developing lung, and its expression under hyperoxic conditions in the neonatal lung.

**Results:**

Fetal (E19), term, 4 day, 8 day and adult rat lung protein and mRNA were analyzed, as well as lungs from neonatal (0–24 hrs) 2 day and 8 day hyperoxic (95% O_2_) exposed animals. mRAGE transcripts in the adult rat lung were 23% greater than in neonatal (0–24 hrs) lungs. On the protein level, rat adult mRAGE expression was 2.2-fold higher relative to neonatal mRAGE expression, and adult sRAGE protein expression was 2-fold higher compared to neonatal sRAGE. Fetal, term, 4 day and 8 day old rats had a steady increase in both membrane and sRAGE protein expression evaluated by Western Blot and immunohistochemistry. Newborn rats exposed to chronic hyperoxia showed significantly decreased total RAGE expression compared to room air controls.

**Conclusion:**

Taken together, these data show that rat pulmonary RAGE expression increases with age beginning from birth, and interestingly, this increase is counteracted under hyperoxic conditions. These results support the emerging concept that RAGE plays a novel and homeostatic role in lung physiology.

## Background

The receptor for advanced glycation end products (mRAGE) belongs to the immunoglobulin superfamily and binds a myriad of ligands such as advanced glycation end products (AGEs)[1], amphoterin [2], and S100/calgranulin [3]. mRAGE has been implicated in the pathology of various disorders, including diabetic atherosclerosis, tumors and inflammation [4]. In contrast, soluble RAGE (sRAGE) acts as a decoy receptor and in the human is formed via alternative splicing [5], generating a novel C-terminal sequence that lacks the mRAGE transmembrane domain. Heparin binding affinity of sRAGE [6] may sustain its presence in the extracellular matrix, acting as a buffer to halt detrimental ligand-mRAGE interactions. mRAGE-ligand interaction has been associated, in particular, with inflammation. In the lung, a recent study has found there to be an increase in RAGE expression in pulmonary pneumonias and smoke-related damage in humans [7]. Notably, in non-pulmonary tissues, mRAGE-ligand interactions have been shown to lead to an upregulation of the receptor [8].

Most tissues express low levels of mRAGE normally and then up-regulate expression of this protein with injury and disease. The lung is a notable exception to this pattern with high levels of RAGE message [9] and protein [10] in the normal adult lung. The high levels of RAGE expression under normal conditions in the lung suggest there may be a novel beneficial homeostatic role for this receptor in this tissue [10].

The full extent of mRAGE and sRAGE expression in the developing neonatal lung has not been examined. mRAGE has been proposed as a marker of type I epithelial cells, which differentiate during lung development [11]. Furthermore, Type II epithelial cells have also been shown to express mRAGE mRNA [12]. The expression of RAGE isoforms and glycosylation patterns in the developing lung remains to be explored. Specifically, there are two potential N-glycosylation sites in both mRAGE [13] and sRAGE [6] that likely influence ligand-receptor affinity [14]. We therefore sought to characterize the expression of RAGE isoforms and their glycosylation patterns in the developing rat lung.

The developing lung undergoes many changes at or near parturition to facilitate the transition from a low fetal oxygen tension to an increased postnatal tension [15]. The etiologies of lung diseases in infants, such as bronchopulmonary dysplasia (BPD), are poorly understood. However, the pathogenesis of BPD has been attributed in part to the inflammation complicated by the requirement for supplemental oxygen therapy, which lead to disrupted alveolar development and damage in the lungs of premature infants [16]. Chronic hyperoxia in newborn rats is an established model for the study of BPD and is associated with increased lung inflammation, increased proteolytic activity and edema [17], which have devastating consequences to the developing lung. Inflammation and oxidative stress can regulate mRAGE expression through reactive oxygen species (ROS) formed as a result of TNF-alpha stimulation in human umbilical vein endothelial cells (HUVEC) [18]. The ability of sRAGE to scavenge detrimental ligands limits binding to mRAGE, and likely confers a beneficial role. A loss of sRAGE or imbalance of sRAGE/mRAGE may contribute to the pathology of BPD. Therefore we sought to examine neonatal rats exposed to hyperoxia to determine its effects on mRAGE and sRAGE expression.

## Results

**Figure 1:**
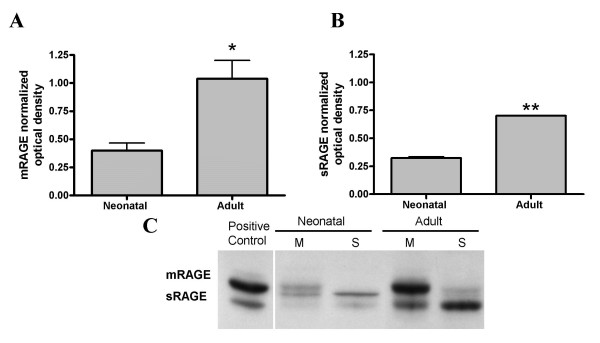
**Expression of mRAGE and sRAGE in neonatal and adult rat lung**. Western blot and subsequent densitometry was performed probing for mRAGE and sRAGE in lung homogenates. Results are normalized to β-actin. **(A) **Neonatal mRAGE expression compared to adult mRAGE (*p < 0.05). **(B) **sRAGE expression in adults compared to that seen in neonates (**p < 0.01). **(C) **Representative Western blot showing sRAGE and mRAGE expression in the neonatal and adult lungs. Notably, a higher molecular weight band is seen in the neonatal soluble preps compared to the adult's and positive control. sRAGE seen in the adult and neonatal membrane preparations represents residual sRAGE remaining in pellet with insoluble proteins during sample preparation.

**Figure 2:**
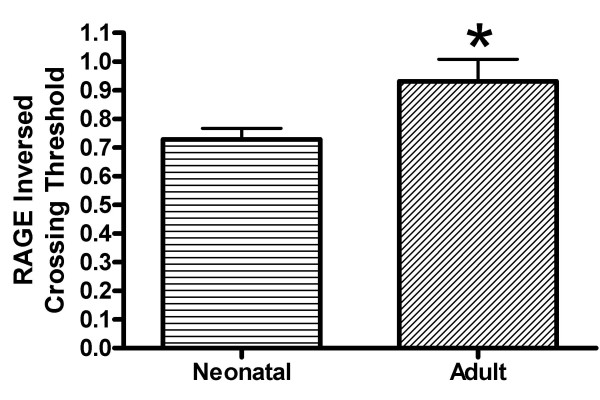
**RAGE mRNA quantified by real-time RT-PCR**. Rat lung RAGE mRNA was quantified by real time PCR amplification. These studies show an increase in adult RAGE mRNA compared to neonates. Results are expressed as inversed crossing-thresholds (CT) normalized to β-actin n = 3 (*p < 0.05).

To examine the developmental expression of RAGE and its isoforms in the lung, mRAGE and sRAGE protein levels were analyzed in rat neonatal (0–24 h) and adult lungs. Western blot analysis revealed a 2-fold increase of mRAGE expression in the adult lung compared to that seen in the neonate (Fig. 1A). Western blot analysis of sRAGE revealed a similar trend in which sRAGE expression was significantly increased in the adult lung compared to the neonatal lung (Fig. 1B). A representative Western blot reveals a molecular weight variance between neonatal sRAGE compared to adult sRAGE (Fig. 1C). This difference in size is likely due to a difference in glycosylation (see below). Real-time RT-PCR amplification revealed a 23% increase in adult mRAGE mRNA compared to neonatal mRNA levels (Fig. 2) (p < 0.05).

**Figure 3:**
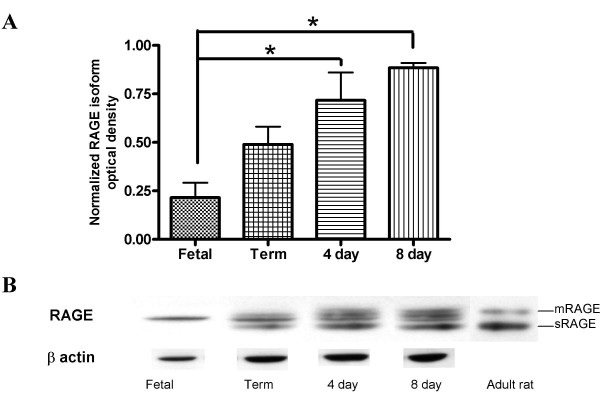
**Developmental expression of mRAGE and sRAGE in the lung**. Lung protein samples were prepared with buffer containing detergent to include both membrane and soluble isoforms of RAGE. **(A) **Densitometry was performed normalizing to β-actin (n = 3). (*p < 0.05) **(B) **Representative Western blot of RAGE isoforms and corresponding β-actin of fetal (E19), term, 4 day, 8 day and adult rat lungs.

**Figure 4:**
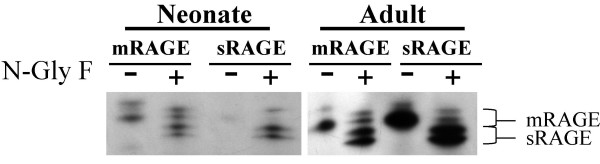
**Deglycosylation of neonatal and adult sRAGE and mRAGE**. Neonatal (0–24 hrs) and adult soluble and membrane rat lung preparations were treated with N-glycosidase F and compared to untreated controls. After deglycosylation neonatal sRAGE bands resolved to match that of the adult sRAGE and neonatal mRAGE matched that of adult mRAGE (n = 2). The two bands seen for both sRAGE and mRAGE reveals a partial and complete deglycosylation.

To further investigate the increase in mRAGE and sRAGE expression seen in development, additional time points were tested. Fetal (E19), term, 4 day, 8 day and adult lungs were collected and analyzed for both mRAGE and sRAGE expression. Western blot analysis revealed a steady increase in mRAGE and sRAGE expression (Fig. 3). Interestingly, the fetal time point (E19) had only a single band as opposed to all postnatal time points that showed bands corresponding to both mRAGE and sRAGE, as well as possible additional glycosylation products of these respective isoforms. Deglycosylation of neonatal and adult mRAGE and sRAGE revealed homology in molecular weight, indicating that alternative glycosylation is likely the cause for the additional bands present in the neonatal samples (Fig. 4) and that the single fetal band was mRAGE.

**Figure 5:**
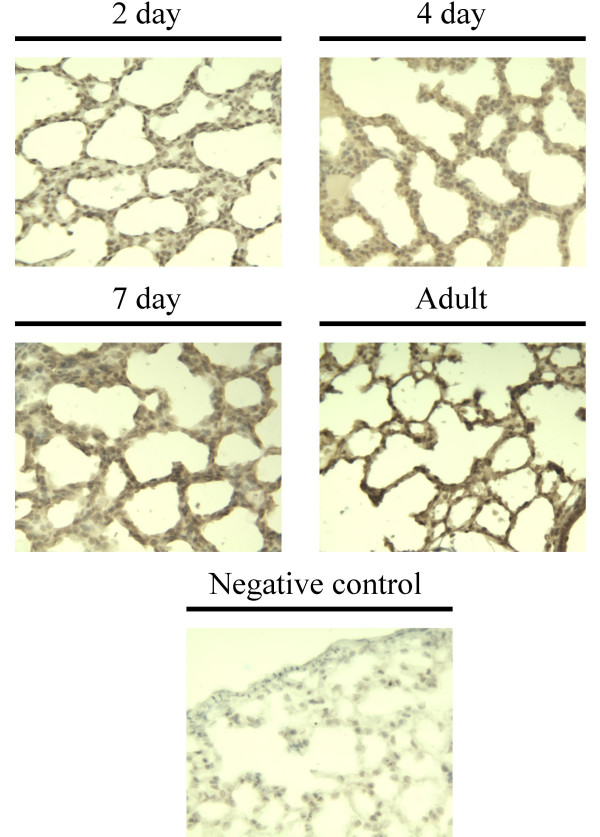
**Immunochemical examination of RAGE expression in lung development**. Two, four, seven day and adult rat lungs were stained for RAGE followed by a hematoxylin counter stain. A gradual increase in positive staining was seen in the alveolar parenchyma with post-natal lung development, with marked staining in type I pneumocytes in the adult.

Immunohistochemical examination of RAGE expression in two, four, seven day and adult rat lungs reveals a gradual rise in marked positive staining with increasing age (Fig. 5). Receptor abundance was predominant in type I pneumocytes.

**Figure 6:**
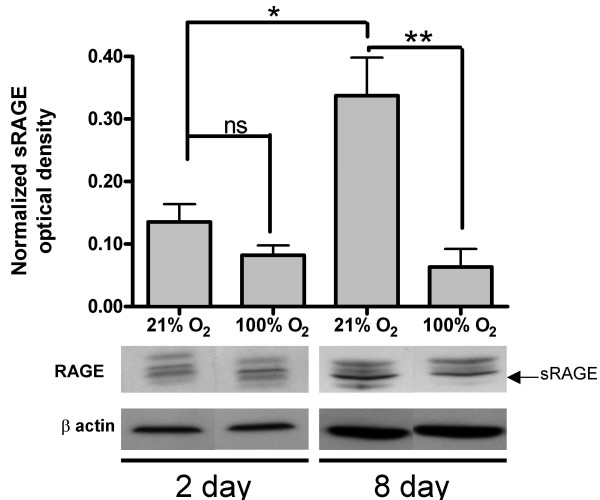
**Effects of hyperoxic exposure on neonatal RAGE expression**. Rat RAGE isoform expression was determined by Western blot and densitometry was performed. Neonatal (0–24 hrs) rats were exposed either to 2 or 8 days of hyperoxia (95% O2) and compared to age matched room air controls (n = 3). Notably a decrease in sRAGE expression is seen after 8 days of hyperoxia (n = 3). (*p < 0.05)

To investigate how neonatal hyperoxia, an established model of BPD, affects mRAGE expression, neonatal rats (0–24 hrs) were exposed to 95% O_2 _for a period of two or eight days. No significant difference in mRAGE or sRAGE expression was evident after 2 days of hyperoxia (Fig. 6). However, after chronic hyperoxia (8 days), sRAGE expression significantly decreased compared to room-air exposed age-matched controls, while mRAGE levels remained constant.

## Discussions and Conclusion

mRAGE and sRAGE expression in normal lung development and in response to hyperoxia were examined. Analysis of the developmental regulation of mRAGE and sRAGE in the lung demonstrates that both neonatal rat mRAGE and sRAGE expressions are lower than found in adult lungs and there is an up-regulation of RAGE isoforms that occurs post-natally. In addition, extended hyperoxic exposures result in a loss of sRAGE in the neonatal rat lung.

Several factors may account for the developmental up-regulation of RAGE in the lung. mRAGE has been identified as a marker of type I epithelial cells [11], and the newborn rat lung is not fully alveolarized. Therefore, the post-natal increase in mRAGE in the neonatal rat lung may reflect ongoing alveolarization characterized by an increase in type I epithelial cells. Furthermore, RAGE expression may be sensitive to oxygen tension [18]. The increase in RAGE isoforms seen in the lung after birth may therefore also be due, in part, to changes in oxygen tension occurring at birth. While immunochemical examination of RAGE can not distinguish between mRAGE and sRAGE due to the inability of the antibody to distinguish these two isoforms, these immunohistochemical studies confirm that the overall level of RAGE is up-regulated during development (Fig. 5). This finding suggests that the increase in RAGE expression does indeed correlate with alveolarization and the increase in type I cells.

A closer look at the soluble and membrane preparations reveals a molecular weight variance between neonatal and adult sRAGE. Interestingly term sRAGE contains a band whose molecular weight does not match that of either the adult sRAGE or neonatal mRAGE. We found that the increase in molecular weight of the neonatal sRAGE is likely due to different glycosylation patterns (see below).

To further assess the expression of mRAGE and sRAGE in the developing lung, additional time-points were explored. Fetal rat lungs express a single RAGE isoform whose molecular weight appears to correspond to the alternatively glycosylated mRAGE expressed in the neonatal lung. This corresponds to its expression in differentiating alveolar epithelial cells as reported previously by Shirasawa *et al*. [11] and Katsuoka *et al*. [12]. Term, four day and eight-day rat lungs demonstrated multiple RAGE bands, which can correspond to different isoforms or glycosylation patterns. It has been elucidated by *Giron et al*[19] that multiple bands due to alternative splicing and glycosylation patterns have been seen in the rat. There are two possible N-glycosylation sites of RAGE. The two sRAGE and mRAGE bands seen after N-glycosydase F treatment reflect a partial deglycosylation (one sugar removed) and complete deglycosylation (both removed). Notably, when neonatal homogenates were deglycosylated, their molecular weights aligned with that of the adults to confirm that alternative glycosylation of the mRAGE and sRAGE is likely responsible for the different sizes of the RAGE bands seen between the early time-points and older rats. Adult rat RAGE expression consists of two distinct bands; the 50 kDa band of mRAGE and the 45 kDa band of sRAGE, consistent with observations in mice [6]. We speculate that alternative glycosylation present in neonates is either absent or present at undetectable levels in the normal adult lung. The alternative glycosylation in the neonate may affect the affinity of the receptor for its ligands. Previous studies looking at amphoterin binding revealed a decrease in radio labeled ligand-receptor binding with deglycosylation [14]. Further work is required to evaluate the whether alternative glycosylation in the neonate affects ligand binding.

Compared to adults, fetal and term lungs contain reduced levels of the sRAGE isoform, which may lead to an increased susceptibility to pathologic insults in the lung. The fact that less sRAGE is present at this stage in development to scavenge detrimental ligands may contribute to the pathologic effects of mRAGE activation, which ultimately leads to NF-kB activation [3]. Under hyperoxic conditions it is known that NF-kB is activated in the neonate but not in the adult [20]. The fact that the mRAGE/sRAGE balance is skewed in neonatal lungs may contribute to this difference in response between adult and neonatal lungs. Alternatively, it has recently been shown that mRAGE may be important in spreading of epithelial cells and that sRAGE can inhibit this epithelial spreading [21]. Thus, the absence of sRAGE at this early time point may promote epithelial spreading of type I cells during alveolar development.

In addition to examining RAGE expression during normal lung development, this study also characterized the effects of hyperoxia on mRAGE and sRAGE expression. One main component of hyperoxic pulmonary injury is inflammation, which can be devastating to the developing lung. Because mRAGE signaling can be highly pro-inflammatory [1] and mRAGE expression is up-regulated in response to inflammation in other tissues, RAGE isoform expression was examined in a hyperoxic setting. Recent work has shown an increase in lung RAGE abundance in pulmonary inflammation caused by smoke-related damage, and various pneumonias; an increase in its ligands has also been shown [7], suggesting a role for RAGE in a novel inflammatory pathway in the lung. However, our results do not show an up-regulation of mRAGE (as it appears unchanged even after chronic hyperoxia), but rather a loss of sRAGE in response to hyperoxic injury. The loss of sRAGE may be due to dilution as a result of edema. Notably, when bronchoalveolar lavage fluid (BALF) was analyzed in an adult model of hyperoxia, sRAGE was detectable by Western blot (not shown), while in normal conditions sRAGE was undetectable in the BALF [10]. Histological examination would be difficult to establish a difference between control and test animals after O_2 _treatment as there is no change in mRAGE and a loss of sRAGE; RAGE antibody detects the variable N-terminal chain of the receptor making it isoform non-specific. However, the Western blot data shows a significant loss of sRAGE, indicating that after hyperoxia, an imbalance in the ratio of sRAGE/mRAGE occurs that may contribute to the pulmonary inflammation seen in a neonatal model of BPD.

The expression of mRAGE in the lung before and after parturition and its subsequent elevated expression in the adult lung, relative to other tissues, support the hypothesis that this protein may play a homeostatic physiological role in this tissue. These experiments have further shown that sRAGE appears to be absent before birth which may help promote type I cell spreading during alveolar development. In addition, sRAGE is found to decrease in response to hyperoxic injury which may promote mRAGE signaling and contribute to further injury in this pathologic condition.

## Methods

### Lung tissue membrane and soluble homogenates

Adult (Pelfreeze) and neonatal (0–24 hrs following parturition) rat lungs were weighed and homogenized in buffer A [50 mM potassium phosphate pH 7.4, 0.3 M potassium bromide, 3 mM diethylenetriamine pentaacetic acid, 0.5 mM phenylmethyl sulfonylfluride], sonicated, and centrifuged at 20,000 g for 20 minutes at 4°C. The supernatant contained the soluble fraction to include sRAGE. The pellet was then resuspended in CHAPS detergent [22,23] vortexed, sonicated and shaken at 4°C for 2 hours. Following incubation for 2 hrs at 4°C, samples were centrifuged at 20,000 g for 20 min at 4°C. This supernatant contained the membrane fraction including RAGE. Western blot analysis was performed as previously described [22-24].

### RNA extraction and quantification

RNA was isolated from adult (Pelfreeze) and neonatal whole lung using the Trizol (Invitrogen) method, as previously described (according to manufacturer's instructions) and analyzed using Light Cycler real-time reverse transcription PCR with SYBR green conjugation (Roche)[25]. Primers were generated for rat RAGE (Sigma); sense primer 5'CTACCTATTCCTGCAGCTTC and rat RAGE anti-sense primer 5'CTGATGTTGACAGGAGGGCTTTCC [19]. For normalization purposes, real-time RT-PCR was performed for β-actin [26]. Cycling was performed as previously described [10] and values expressed as inversed crossing-threshold.

### Lung tissue total protein homogenates

Lung tissue from fetal, term, four day, eight day and adult rats was homogenized on ice in lysis buffer containing 50 mM Tris pH 7.6, 3% Igepal, 150 mM NaCl, 1 mM MgCl_2_, and 5 mM EDTA. Lysis buffer also included 1:20 protease inhibitors 1 mM 1, 10 phenanthroline, 2 mM 3,4 diisocoumarin, and 0.4 mM trans-epoxysuccinyl-L-leucylamideo(4-guanidino)butane (E-64). Homogenates were assayed for protein abundance and Western Blot performed.

### Immunohistochemistry of RAGE in the developing rat lung

Rat lung tissue was formalin fixed and paraffin embedded, from which 5 micron sections were obtained. Antigen retrieval was accomplished with a 40 min boil in Tris/Urea buffer, (Tris-HCl, 5% urea, pH 6.0) and cooled for 20 minutes at room temperature (RT). Endogenous peroxidase activity was blocked with 3% H_2_O_2 _for 10 minutes at RT. Immunolabeling for RAGE was performed using a rabbit anti-RAGE primary antibody (1:100) (Santa Cruz) prepared in 1%BSA/PBS and detected using HRP conjugated secondary (BioGenex) followed by color development in 3,3-diaminobenzidine (DAB) and counterstained with hematoxylin as previously described [23].

### Hyperoxic Exposures

Paired timed pregnant Sprague-Dawley rats (Charles River Laboratories, Charles River, NJ) were obtained during late gestation. Within 24 hours of delivery, the litters were split evenly between two nursing dams and assigned randomly to either air or 95% oxygen exposure for up to one week. The dams were alternated every 24 hours between the air and oxygen-exposed litters to prevent maternal oxygen toxicity. After the exposure period, the rats were euthanized with intraperitoneal pentobarbital (150 mg/kg), lungs were perfused with 10 cc saline to remove blood and lung tissue was flash-frozen in liquid nitrogen and homogenized for total protein. All animal procedures were approved by the Institutional Animal Care and Use Committee [27,28].

### Deglycosylation

Semi-purified RAGE and sRAGE were obtained by microtube chromatography of 50 μg of protein from each sample utilizing Affi-Gel Heparin Gel (Bio-rad) to which sRAGE is known to bind [6]. Samples were then concentrated using Microcon Citrifugal filter device (Millipore) and centrifuged at 12,500 g for 20 min. Deglycosylation of RAGE and sRAGE was performed on these concentrated samples using N-Glycosidase F (2 μl for 100 U)(Calbiochem) as previously described [6]. Western blot analysis was performed to determine glycosylation pattern, as previously described [10].

### Statistical Analysis

Results were analyzed using a Student's-T test for paired samples and one-way ANOVA followed by Tukey's multiple comparison test to determine significance among a group, values of p < 0.05 were deemed significant.

## Authors' contributions

PPL carried out PCR and Western blot analysis, performed immunohistological staining and wrote the manuscript. LH provided direct supervision, aided in the projects analyses and revised the manuscript. JE provided input in the projects design and revised the manuscript. ENG and BLG designed and performed the hyperoxic experiments and provided the tissue, as well as providing critical review of the manuscript. TDO conceived of the study and participated in its coordination, interpretation, and writing. All authors read and approved the final manuscript.
